# The efficacy and safety of iguratimod treatment in stable systemic lupus erythematosus: a preliminary prospective cohort study

**DOI:** 10.3389/fmed.2026.1810291

**Published:** 2026-06-01

**Authors:** Lei Zhang, Meng Chen, Bing Shen, Zhengdong Shen, Yongliang Chu, Ruoxin Huang

**Affiliations:** 1 The Second Affiliated Hospital of Guangzhou University of Chinese Medicine, Guangdong Provincial Hospital of Chinese Medicine, The Second Clinical Medical College, Guangzhou University of Chinese Medicine, Guangzhou, China; 2 Department of Rheumatology, Zhuhai Branch of The Second Affiliated Hospital of Guangzhou University of Chinese Medicine, Zhuhai, China; 3 Neher’s Biophysics Laboratory for Innovative Drug Discovery, State Key Laboratory of Mechanism and Quality of Chinese Medicine, Macau University of Science and Technology, Macau, Macau SAR, China

**Keywords:** efficacy, explore, flare, iguratimod (T-614), systemic lupus erythematosus

## Abstract

**Objectives:**

To evaluate the efficacy and safety of iguratimod as maintenance therapy in patients with stable systemic lupus erythematosus (SLE).

**Methods:**

Eligible participants were adults with stable SLE (SLEDAI-2K ≤4 for ≥12 weeks) and on stable standard-of-care medication. Patients treated with iguratimod (iguratimod group) were matched to those not receiving iguratimod (control group) using prospectively frequency matching at a 1:2 ratio. The coprimary end points were the flare rate at week 52 and the time to first flare.

**Results:**

This prospective cohort study consecutively screened 220 patients, of whom 52 meeting the inclusion criteria were enrolled. The final analysis included 16 patients in the iguratimod group and 31 in the control group. In the coprimary analysis, The flare rates at week 52 were 37.5% in the iguratimod group and 29% in the control group. Iguratimod was associated with a non-significant, numerically higher relapse risk versus control (HR = 1.45, 95% CI 0.57 to 3.74), with sensitivity analyses confirming the direction of this trend. No significant between-group differences were observed in the changes of secondary endpoints. Regarding safety, iguratimod was well-tolerated, and no serious adverse events were reported.

**Conclusion:**

This cohort study of 52 stable SLE patients found that the 52-week flare risk did not differ significantly between those exposed to iguratimod and those who were not. However, these null findings should be interpreted with caution and are not evidence of equivalence.

## Introduction

Systemic lupus erythematosus (SLE) is a multisystem autoimmune disease (1). The condition is characterized by high heterogeneity in both clinical manifestations and disease progression, with a relapse-remission pattern being its natural course (2). Importantly, each disease flare is potentially associated with increased damage accrual and a higher risk of adverse outcomes (3, 4). Consequently, maintaining long-term disease stability constitutes a fundamental principle in the management of SLE (5). Although substantial progress has been made in the maintenance therapy for SLE, approximately 70% of patients still experience a relapsing-remitting course (6). Therefore, unmet therapeutic needs remain a central challenge in SLE care (7).

Iguratimod (IGU), a novel immunomodulatory agent, has demonstrated promise in the treatment of autoimmune diseases, including rheumatoid arthritis (RA). In two Phase III clinical trials, iguratimod (50 mg/day) was compared with placebo, methotrexate (MTX), and sulfasalazine (SASP) for efficacy and safety. The results indicated that iguratimod was significantly more effective than placebo and had comparable efficacy to MTX and SASP, with a favorable safety profile (8–10). Currently, iguratimod is approved in Northeast Asia for the treatment of RA patients with an inadequate response to MTX. Furthermore, in patients with primary Sjögren’s syndrome (pSS), the combination of iguratimod and glucocorticoids has been shown to reduce clinical symptoms and disease activity more effectively than hydroxychloroquine (HCQ) plus glucocorticoids, while also improving exocrine gland function (11). Previous small-scale observational studies have suggested the potential utility of iguratimod in treating refractory lupus nephritis (12, 13). Mechanistically, preclinical studies have shown that iguratimod reduces the expression of BAFF, IL-6, IL-17A, and IL-21 in female MRL/lpr mice, modulates T and B cell function, and diminishes B-cell infiltration in the kidneys (14). Similarly, in a pristane-induced lupus nephritis model, iguratimod restored the balance between Th17 and Treg cells, ameliorating renal pathology (15).

Based on these evidences, this study aimed to evaluate the efficacy and safety of iguratimod in patients with stable SLE.

## Materials and methods

### Study design

**FIGURE 1:**
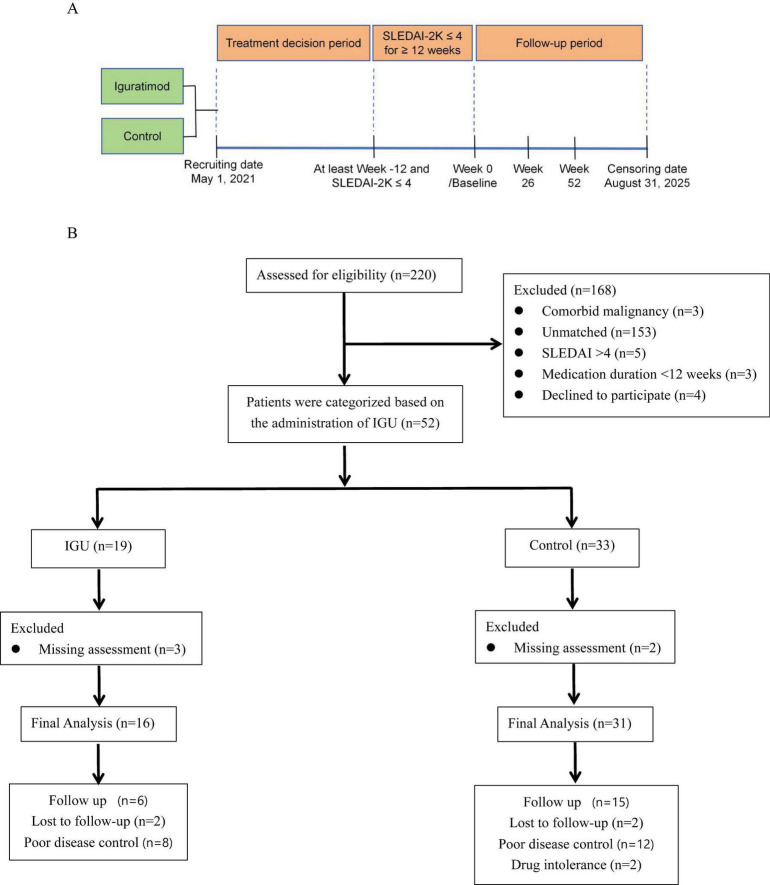
Study design **(A)** and patient flow diagram through week 52 **(B)**. IGU, iguratimod.

This prospective cohort study was conducted to evaluate the efficacy and safety of iguratimod in patients with stable SLE over a follow-up period of at least 1 year (Figure 1A). Participants were consecutively recruited from the rheumatology outpatient clinic of the Second Affiliated Hospital of Guangzhou University of Chinese Medicine (Guangdong Provincial Hospital of Chinese Medicine) between May 2021 and April 2024. Data on demographics, clinical symptoms, disease assessments, medications, and adverse events were collected. Patients were followed up at intervals ranging from 1 to 6 months, based on their clinical condition.

### Participants

Participants were eligible for the study if they met the following criteria: (1) fulfilled the 2012 Systemic Lupus International Collaborating Clinics (SLICC) classification criteria for systemic lupus erythematosus (16); (2) aged 18 years and above; (3) had a stable SLE Disease Activity Index 2000 (SLEDAI-2K) score of ≤4 for at least 12 weeks prior to enrollment; (4) maintained stable doses and types of immunosuppressants, immunomodulator, and/or glucocorticoids for at least 12 weeks; (5) had a body weight of no less than 40 kg.

Individuals were excluded from participation for any of the following reasons: (1) presence of severe comorbidities, including heart failure (New York Heart Association Class III or IV), respiratory failure, renal insufficiency (estimated glomerular filtration rate ≤30 mL/min/1.73 m^2^), hepatic insufficiency (alanine aminotransferase or aspartate aminotransferase levels ≥2 times the upper limit of normal), or severe hypertension; (2) prior use of biologic agents or small-molecule targeted therapies; or having received treatments such as plasmapheresis, immunoadsorption, hemodialysis, or mesenchymal stem cell transplantation; (3) history of malignancy, with the exception of cured carcinoma *in situ* or basal cell carcinoma; (4) pregnancy or lactation. Written informed consent was obtained from all participants prior to their enrollment in the study.

### Procedures

#### Study groups and matching

Patients treated with iguratimod (iguratimod group) were prospectively frequency-matched at a 1:2 ratio to those not receiving iguratimod (control group). Matching variables included sex, age, organ systems involved and duration of maintenance therapy. The matching procedure was performed as follows: using the cumulative distribution of the already-enrolled iguratimod group across strata of the matching variables as the reference, control subjects were concurrently and consecutively recruited to maintain a 1:2 case-to-control ratio within each stratum. Enrollment of controls for a specific stratum was temporarily suspended once the 1:2 ratio was reached and resumed only when additional iguratimod patients were enrolled in that stratum.

#### Treatment and follow-up protocol

Following enrollment, all patients continued their treatment regimens. During the follow-up period, glucocorticoid dosages could be tapered based on clinical disease activity assessments; however, the regimens of concomitant immunosuppressants were to remain unchanged. Safety and laboratory monitoring included complete blood counts, liver function tests, and renal function tests, which were performed at intervals of 1–6 months. Disease activity assessments, including 24-h urinary protein, anti-double-stranded DNA (anti-dsDNA) antibodies, serum complement 3 (C3), and complement 4 (C4) levels, were measured at baseline and every 6 months thereafter, or at the time of premature study withdrawal.

The study protocol was approved by the Ethics Committee of Guangdong Provincial Hospital of Chinese Medicine (Approval No. BE2021-023-01) and was subsequently registered in the Medical Research Registration and Filing Information System of China (Registration ID: MR-44-22-021595).

### Outcomes

Coprimary end points were the flare rate at week 52 and the time to first SLE flare. The flare defined as a composite of: (1) an increase in SLEDAI-2K score by ≥3 points from baseline; (2) the emergence of new clinical manifestations of SLE or worsening of pre-existing symptoms, necessitating the re-initiation or escalation of therapy (including glucocorticoids, non-steroidal anti-inflammatory drugs (NSAIDs), HCQ or immunosuppressants).

Secondary endpoints included: (1) changes from baseline in daily glucocorticoid dosage and SLEDAI-2K score at week 52; (2) changes from baseline in serum complement and immunoglobulin levels at week 26; (3) drug retention rate and the proportion of patients maintaining/achieving a clinical SLEDAI (cSLEDAI) = 0, and glucocorticoid discontinuation (prednisone ≤5 mg/day) by the censoring date (on or before 31 August 2025).

### Sample size

Given the exploratory and observational nature of this study aimed at generating hypotheses, a formal sample size calculation was not performed. The sample size was determined by the availability of eligible patients who met the inclusion criteria at our institution during the designated study period.

### Statistical analysis

Continuous variables with a normal distribution are presented as mean ± standard deviation (SD), while those with a non-normal distribution are expressed as median (interquartile range, IQR). Categorical variables are summarized as numbers (percentages) and were compared using the Chi-square test or Fisher’s exact test, as appropriate. For between-group comparisons, independent samples *t*-tests were used for normally distributed data, and the Mann-Whitney U test was employed for non-normally distributed data. A two-sided value of *P* ≤ 0.05 was considered statistically significant. Changes in continuous outcomes over time were assessed using an analysis of covariance (ANCOVA) model, adjusting for baseline values, and a linear mixed model was applied for analyzing repeated measures data. The time to flare data were visualized with Kaplan-Meier curves and compared between groups using the log-rank (Mantel-Cox) test. For the analysis of serologic indicators at the 26-week time point, the last observation was carried forward (LOCF) for patients who altered their treatment regimen prior to the scheduled 26-week visit. Missing laboratory data for immunoglobulins (IgA, IgG, IgM) were handled using multiple imputation. Twenty imputed datasets were created with 50 iterations, incorporating prognostic and outcome-related covariates into the imputation model (e.g., disease activity, organ systems involved and disease duration). Pooled estimates were derived according to Rubin’s rules.

To assess the robustness of the primary findings, a sensitivity analyses was performed: the time to first flare was conducted using a modified intention-to-treat (mITT) approach. In this analysis, all patients who were lost to follow-up or deviated from the original treatment protocol for any reason, were considered as having experienced a treatment failure. All statistical analyses were performed using IBM SPSS Statistics version 27.0 and GraphPad Prism version 8.0.

## Results

### Baseline patient characteristics

Among 220 screened patients with SLE, 52 were ultimately enrolled in this study. Based on their treatment regimen, patients were categorized into an iguratimod group (*n* = 19) and a control group (*n* = 33). Following enrollment, three patients from the iguratimod group and two from the control group were excluded from the final analysis due to missing SLEDAI-2K data. Consequently, the final analysis included a total of 47 patients, with 16 assigned to the iguratimod group (Table 1) and 31 to the control group (Supplementary Table 1). A flowchart of the study is presented in Figures 1B. A comparative analysis of their baseline characteristics is summarized in Table 2. At baseline, the mean age of patients was 43.6 (±10.6) years in the iguratimod group and 42.2 (±10.7) years in the control group. The mean body weight was 54.8 (±7.3) kg and 57.2 (±7.3) kg, respectively. All enrolled patients were female. The median follow-up duration was 53.5 (IQR: 21.5–154) weeks in the iguratimod group and 108 (IQR: 42–128) weeks in the control group. In the iguratimod group, all patients were on a regimen of iguratimod and HCQ; 5 of these patients also received a concomitant immunosuppressive agent, whereas the remaining 11 received the two drugs without an additional immunosuppressive agent. No significant differences were observed between the two groups regarding autoantibody profiles, glucocorticoid dosage, duration of stable disease, duration of stable immunosuppressant or HCQ) therapy, SLEDAI-2K scores.

**TABLE 1:** Baseline characters and outcomes of the IGU group.

Patient no.	Age	Sex	Duration* (Y)	Antibodies^Δ^	Organ involvement	Reason for drug change and regimen^Σ^	IS AND IMM^#^	Pre-enrollment stability period (w)^&^	Prednisone dose^§^	SLEDAI-2K**	Duration of IGU (w)^$^	Outcome at the last visit	Reason for discon-tinuing
1	61	F	0.5	SSA, RO-52	Mucocutaneous, musculoskeletal	Arthritis; replaced MTX	HCQ	12	5	4	32	Arthritis flare-up; prednisone discontinued, added telitacicept	Arthritis symptoms improved without complete resolution.
2	47	F	5	SSA, RO52	Mucocutaneous, musculoskeletal	MTX intolerance; replaced MTX	HCQ	120	0	0	208	Stable condition	Follow-up
3	39	F	2	SSA, RO-52, Sm, MDA5,U1-RNP, Rib-P	Mucocutaneous, Musculoskeletal	Raynaud’s phenomenon with rash; add-up	CTX, HCQ	16	7.5	0	225	Rash resolved; CTX tapered off; prednisone reduced to 5mg	Follow-up
4	45	F	3	Histone, kinetochore, nucleosome	Renal	Abnormal lab: urinary pathologic casts, low complement; replaced AZA	HCQ	12	7.5	0	53	Normal lab results; prednisone reduced to 5mg	Lost to follow-up
5	37	F	3	SSA, RO-52, SSB, dsDNA, Histone	Neurological, Musculoskeletal	Arthritis; add-up	MMF, HCQ	20	7.5	2	191	Arthritis resolved; prednisone reduced to 5 mg	Follow-up
6	52	F	21	Sm, SSA	Mucocutaneous	Rash, CsA intolerance; replaced CsA	HCQ, MTX	24	10	3	52	Rash resolved; prednisone reduced to 5 mg	Poor compliance despite stable condition
7	30	F	1	U1-RNP, Sm, SSA, RO-52,	Musculoskeletal	Arthritis; add-up	HCQ	12	10	0	136	Arthritis flare-up (relapse 9 months after prednisone taper)	Arthritis flare-up
8	49	F	23	JO-1, SSA, RO-52, U1-RNP, Sm,	Musculoskeletal, respiratory	Arthritis with elevated creatine kinase; replaced AZA	MTX, HCQ	12	7.5	2	54	Arthritis flare-up; switched to MMF; prednisone increased to 15 mg	Arthritis flare-up
9	33	F	16	SSA, histone	Mucocutaneous	Rash; replaced T	HCQ	12	0	4	8	Worsening rash; added 10 mg prednisone and azathioprine	Worsening rash
10	55	F	4	Sm, SSA, SSB	Musculoskeletal, neurological	Arthritis; add-up	HCQ	20	5	0	26	Arthritis flare-up; switched to MTX	Arthritis flare-up with UTI
11	51	F	2	Sm, SSA, U1RNP, Scl-70	Musculoskeletal, mucocutaneous	Arthritis; replaced leflunomide due to intolerance	HCQ	12	0	0	104	Arthritis resolved	Follow-up
12	57	F	12	Sm, SSA, RO-52, u1-RNP	Musculoskeletal	Arthritis; replaced T	HCQ	20	0	2	58	Arthritis resolved	Follow-up
13	38	F	19	Sm	Mucocutaneous, renal	Rash; add-up	HCQ	24	0	0	160	Rash resolved	Follow-up
14	30	F	5	Sm	Mucocutaneous	Rash; add-up	HCQ	20	5	2	8	Worsening rash; prednisone increased to 10 mg	Worsening rash
15	26	F	4	SSA, SSB, RO-52	Hematologic, musculoskeletal	Thrombocytopenia; replaced CsA	HCQ	15	5	1	20	Worsening Thrombocytopenia; prednisone increased to 30 mg	Worsening Thrombocytopenia
16	47	F	13	Sm, U1RNP, SSA	Musculoskeletal, mucocutaneous	Rash; add-up	T, HCQ	20	10	2	14	Rash flare-up (relapse 3 months after prednisone taper); switched to MMF	Rash flare-up

*Disease duration was defined as the time from the onset of initial clinical symptoms to study enrollment.

^Δ^Autoantibody status at the time of enrollment. All enrolled patients had a positive antinuclear antibody (ANA) titer of > 1:100 (reference value: 1:100).

^Σ^Symptoms were considered present if they were new or relapsing.

^#^Use of disease-modifying antirheumatic drugs (DMARDs) and immunomodulators at baseline, with stable doses for at least 12 weeks prior to enrollment.

^&^Duration of stable disease activity, defined as the time from when the SLEDAI-2K score was first documented to be ≤ 4 until enrollment.

^§^Dosage of glucocorticoids at baseline, which had been stable for at least 12 weeks prior to enrollment.

**SLEDAI-2K score at baseline.

^$^The observation period spanned from the date of study enrollment to the date of the last follow-up visit. SLEDAI-2K, SLE Disease Activity Index 2000; IS, immunosuppressant; Imm, immunomodulator; HCQ, hydroxychloroquine; IGU, iguratimod; AZA, azathioprine, TGP, total glucosides of white paeony; MMF, mycophenolate mofetil; CsA, cyclosporin A; MTX, methotrexate; CTX, cyclophosphamide; T, thalidomide; UTI, urinary tract infections.

**TABLE 2:** Baseline characteristics of two groups.

Characteristics	Iguratimod (*n* = 16)	Control (*n* = 31)	*P*
Age (y)	43.6 ± 10.6	42.2 ± 10.7	0.678
Sex, female (%)	16 (100)	31 (100)	–
Time since SLE onset (y)	4.5 (2.3–15.3)	2.0 (1.0–9.0)	0.117
Weight (kg)	54.8 ± 7.3	57.2 ± 7.3	0.272
Pre-enrollment stability period (w)	18.0 (12.0–20)	20.0 (12.0–44)	0.188
Autoantibodies (%)			
Sm	9 (56.3)	21 (67.7)	0.437
dsDNA	1 (6.3)	3 (9.7)	1.000
Rib-P	1 (6.3)	6 (19.4)	0.396
SSA	13 (81.3)	18 (58.1)	0.112
SSB	3 (18.8)	5 (16.1)	1.000
Nuc	1 (6.3)	5 (16.1)	0.648
Organ involvement (%)			
Musculoskeletal	11 (68.8)	26 (83.9)	0.274
Mucocutaneous	9 (56.3)	20 (64.5)	0.581
Hematology	1 (6.3)	7 (22.6)	0.234
Renal	2 (12.5)	5 (16.1)	1.000
respiratory	1 (6.3)	2 (6.5)	1.000
Neurological	2 (12.5)	1 (3.2)	0.264
Pre-baseline maintenance therapy duration (w)	18.0 (12.0–20.0)	20.0 (12.0–44.0)	0.188
Concomitant medications			
Prednisolone (mg)	5.0 (0.0–7.5)	5.0 (5.0–10.0)	0.601
No glucocorticoids (%)	5 (31.3)	6 (19.4)	0.472
Patients receiving HCQ plus an immunosuppressant or IGU (%)	11 (68.8)	26 (83.9)	0.270
Disease activity score			
cSLEDAI = 0	10 (62.5)	18 (58.1)	0.769
SLEDAI-2K	1.5 (0.0–2.0)	2.0 (0.0–4.0)	0.206

### Efficacy

#### Flare rate

**FIGURE 2:**
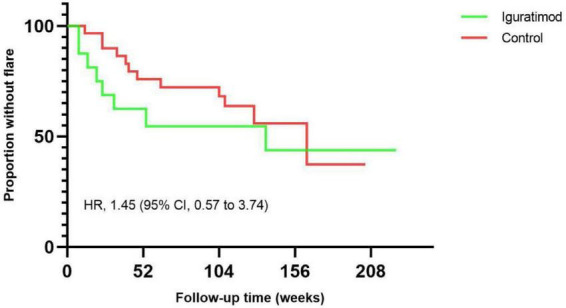
The Kaplan-Meier survival curve showing the proportion of systemic lupus erythematosus patients of the two groups experiencing a flare over study period. The proportion of patients of the two groups experiencing a flare over study period.

In the coprimary analysis, disease flare occurred in 6 (37.5%) and 9 (29%) patients in the iguratimod and control groups, respectively, with no significant difference observed in the flare rate between the two groups at the 52-week follow-up (Table 3). As the flare event rate was low, precluding the estimation of median time to flare, the primary comparison of relapse risk is expressed as a hazard ratio (HR). Kaplan-Meier analysis showed iguratimod group exhibited a higher risk of flare, the difference between the groups was not statistically significant (HR = 1.45, 95% CI 0.57 to 3.74, *P* = 0.403) (Figure 2). The sensitivity analysis for treatment failure did not suggest a significant difference between the iguratimod and control groups as well (HR = 1.39, 95% CI 0.61 to 3.19; *P* = 0.408) (Supplementary Figure 1).

**TABLE 3:** Categorical variables endpoints in two groups.

Endpoint	Iguratimod (*n* = 16)	Control (*n* = 31)	*χ* ^2^	*P*
Relapse rate (%)^#^	6 (37.5)	9 (29.0)	0.348	0.555
cSLEDAI = 0 (%)*	6 (37.5)	15 (48.4)	0.506	0.477
Drug retention rate (%)*	7 (43.8)	15 (48.4)	0.091	0.763
cSLEDAI = 0 at final follow-up	Iguratimod (*n* = 6)	Control (*n* = 15)		
Attainment of cSLEDAI = 0 from > 0*	0 (0.0)	7 (46.7)	–	0.055
Among initial GC users	Iguratimod (*n* = 11)	Control (*n* = 25)		
Successful GC reduction (%)^&^*	2 (18.2)	10 (40.0)	–	0.268

^#^At 52 weeks. *Most recent visit at or before censoring date.

^&^Prednisolone ≤ 5 mg. GC, glucocorticoid; cSLEDAI, clinical SLEDAI.

#### SLEDAI and glucocorticoid dose

In an analysis of patients transitioning from a cSLEDAI >0 to cSLEDAI = 0 by the most recent visit, this endpoint was achieved in 0 of 6 and 7 of 15 (46.7%) patients in the iguratimod and control groups, respectively (*P* = 0.055) (Table 3). Among patients who remained in follow-up and were using glucocorticoids at baseline, successful reduction to a prednisolone dosage of ≤5 mg/day was accomplished in 2 of 11 (18.2%) in the iguratimod group, compared with 10 of 25 (40.0%) in the control group (Table 3). Notably, disease flare following glucocorticoid reduction occurred in two patients in the iguratimod group and five in the control group (Table 1 and Supplementary Table 1).

The assessment of changes in SLEDAI-2k and glucocorticoid dosage over time, via a linear mixed-effects model, revealed that baseline SLEDAI-2K and glucocorticoid dosage—but not treatment group or its interaction with time—were significant predictors of outcome (Table 4 and Supplementary Table 2).

**TABLE 4:** Linear mixed-effects model analysis of the SLE Disease Activity Index 2000 (SLEDAI-2K) and glucocorticoid dosage at 26 and 52 weeks.

Variable	Estimate	Std. error	Sign.	95% CI	
				Lower bound	Upper bound
A SLEDAI-2K Group (IGU vs. control)^a^	0.165	0.319	0.607	−0.473	0.803
B Glucocorticoid dosage Group (IGU vs. control)^a^	−0.311	0.772	0.689	−1.855	1.233

^a^Adjusted for baseline values and disease relapse status. IGU, iguratimod.

#### Serologic indicators

Data on immunoglobulins were missing in both groups, with two cases in the iguratimod group and three in the control group. No significant differences were observed in the raw values of C3 (HLMD: 0.02; 95% CI: −0.02, 0.04), C4 (HLMD: 0.01; 95% CI: −0.03, 0.07), IgA (HLMD: −0.03; 95% CI: −0.22, 0.05), (HLMD: 0.33; 95% CI: −1.89, 0.56), or IgM (HLMD: 0.06; 95% CI: −0.10, 0.04) between the iguratimod and control groups. To account for potential confounding factors, an analysis of covariance (ANCOVA) was performed, adjusting for baseline values and disease relapse status. The ANCOVA results confirmed the initial findings, demonstrating no statistically significant between-group differences in the levels of C3, C4, IgA, IgG, or IgM after adjustment (Supplementary Table 3).

### Drug retention

As of 31 August 2025, seven patients in the iguratimod group remained on the original treatment regimen, and six were still under active follow-up. Notably, one of these patients (No.1), despite experiencing symptomatic improvement after initiating iguratimod, had persistent arthritis that did not completely resolve. In the control group, 15 patients continued the original regimen, with all remaining in follow-up.

#### Safety

In the control group, two patients discontinued the treatment due to recurrent oral ulcers and gastrointestinal discomfort, respectively, and switched to alternative medications. Regarding infections, one case of recurrent urinary tract infection (UTI) occurred in the iguratimod group, while one case of recurrent UTI and two cases of pulmonary infection were reported in the control group. Among these three patients with infections, symptoms resolved following oral antibiotic therapy combined with a switch to another immunosuppressant, and one patient recovered with oral antibiotics alone. No serious adverse events were reported in either group during the follow-up period (Table 5).

**TABLE 5:** Side effects during follow-up.

Side effects	Iguratimod (*n* = 16)	Control (*n* = 31)	χ^2^	*P*
Adverse reaction (%)	1 (6.3)	5 (16.1)	–	0.648
Dropped due to adverse reactions (%)	0 (0.0)	2 (6.5)	–	0.541

## Discussion

In the present study, we explored a previous unknown feasibility of iguratimod as a combination therapy for maintenance treatment in patients with stable systemic lupus erythematosus (SLE). The rationale for exploring iguratimod in SLE is supported by previous small-scale observational studies indicating its efficacy in refractory lupus nephritis (12, 13). An ongoing randomized controlled trial is further investigating iguratimod in active lupus nephritis (17). However, data on the efficacy and safety of iguratimod during the stable phase of SLE have been lacking. Our prospective controlled study was designed to address this gap.

Reported flare rates in SLE maintenance therapy provide a relevant context for our findings. A 6-month follow-up of patients maintained on mycophenolate mofetil (MMF) after remission documented a relapse rate of 32% (18), while the control group (HCQ or MMF) in a 52-week belimumab maintenance trial had a rate of 22.3% (19). In our study, the 52-week relapse rates were 37.5% for the iguratimod group and 29% for the matched control group. But, several aspects of our study design complicate direct comparison with most other cohorts. First, a mix of patients were enrolled who could be classified as being in DORIS remission (cSLEDAI = 0) (20) or in APLC-defined LLDAS (SLEDAI-2K ≤4) (21). Unfortunately, our small sample size and the lack of Physician Global Assessment (PGA) data rendered a stratified analysis statistically unfeasible. Second, the required duration of maintained low disease activity (SLEDAI ≤ 4) in our study was shorter than in some other studies (22). Third, To better evaluate the role of iguratimod in stable SLE, we established a matched control group.

Our coprimary finding was that there was no significant difference in the flare rate between the iguratimod and control groups. In this exploratory analysis, the point estimate for relapse risk was numerically higher in the iguratimod group (HR = 1.45), a trend that should be interpreted with extreme caution given the wide confidence interval and small sample size. Sensitivity analyses did not alter this directional trend. In this context, a post-hoc power calculation was performed based on the observed sample size (19 vs. 33). Assuming a 30% flare rate in unexposed patients, a 20% absolute risk reduction in the exposed group, and a two-sided alpha of 0.05, the achieved power was approximately 31%. This low power indicates a high risk of type II error; therefore, the null findings should be interpreted with caution and are not evidence of equivalence.

Since the introduction of the treat-to-target (T2T) strategy for SLE by an international task force (23), substantial evidence has supported its feasibility and clinical value (24). Achieving and maintaining treatment targets remains the cornerstone of SLE management, as prolonged disease stability is associated with reduced flare rates and damage accrual. Furthermore, sustaining remission or LLDAS over the long term leads to improved prognosis (25, 26). However, LLDAS is still associated with a higher risk of damage accumulation compared to complete remission (25). Therefore, striving for and maintaining remission is critical for minimizing adverse long-term outcomes. Throughout our study, the treating physicians adhered to the core principle of reducing or discontinuing glucocorticoids while maintaining disease stability, particularly in patients without major organ involvement. This approach aligns with the 2025 ACR Guideline for the treatment of SLE regarding glucocorticoid taper (27).

Guided by the 2021 DORIS remission criteria, we specifically focused on glucocorticoid dosage and cSLEDAI scores (20). In our cohort, five patients in the iguratimod group were not using glucocorticoids at baseline, of whom four remained in follow-up without flare. In the control group, six patients were glucocorticoid-free at baseline, with five remaining in follow-up. This observation is consistent with the established view that low glucocorticoid use is a key factor in achieving sustained stability in SLE (28). It is worth noting that among the six patients in the iguratimod group who completed follow-up without flaring, only 2 (33.3%) were using glucocorticoids at baseline. In contrast, among the 15 non-flaring patients in the control group, 10 (66.7%) were on glucocorticoids at baseline. A similar pattern was observed for cSLEDAI: among the six non-flaring iguratimod patients, none of them had a baseline cSLEDAI >0, compared to 7 out of 15 (46.7%) non-flaring controls. These observations also underscore the need for cautious interpretation regarding the efficacy of iguratimod as maintenance therapy.

One of this study’s strengths is the study employed a 1:2 matched cohort design, endeavoring to mitigate selection bias by employing a prospectively frequency matched control group. Second, the study population consisted of patients with stable disease, reflecting a common clinical scenario in long-term SLE management. In addition, this is not a controlled randomized study, but comprises real-world data as seen in clinical practice.

There are some limitations in this study that should be considered when interpreting the findings: (1) The primary limitations are the small sample size and potential selection bias, which—coupled with the small number of flares—likely reduced statistical power to detect significant differences in outcomes. (2) The enrolled patients were characterized by an older age at disease onset and a predominance of musculoskeletal, mucocutaneous, and cutaneous lupus erythematosus (CLE) involvement. Such a clinical profile is generally associated with a more benign disease course and a lower risk of flare (22, 29), which may have led to an overestimation of iguratimod’s efficacy. (3) As a single-center study conducted in an East Asian population, where SLE often presents with a less severe phenotype compared to other ethnicities (30), the generalizability of our findings to other populations may be limited. (4) The median follow-up duration was relatively short, potentially missing long-term outcomes and late flares. (5) The study did not consistently use LLDAS/remission criteria for enrollment and evaluation, nor did it account for background immunosuppressants in matching—particularly glucocorticoid dose. This is critical because patients with SLEDAI ≤ 4 on no steroids versus 10 mg/day prednisolone have markedly different flare risks, despite both meeting the current definition of “stable” SLE.

## Conclusion

This cohort study of 52 stable SLE patients found that the 52-week flare risk did not differ significantly between those exposed to iguratimod and those who were not. However, these null findings should be interpreted with caution and are not evidence of equivalence.

## Data Availability

The original contributions presented in this study are included in this article/Supplementary material, further inquiries can be directed to the corresponding authors.

## References

[1] Siegel CH Sammaritano LR. Systemic lupus erythematosus: a review. *JAMA.* (2024) 331:1480–91. 10.1001/jama.2024.2315 38587826

[2] Wilhelm TR Magder LS Petri M. Remission in systemic lupus erythematosus: durable remission is rare. *Ann Rheum Dis.* (2017) 76:547–53. 10.1136/annrheumdis-2016-209489 27558987

[3] Kandane-Rathnayake R Milea D Louthrenoo W Hoi A Golder V Cho J et al. Longitudinal associations of flare and damage accrual in patients with systemic lupus erythematosus. *Lupus Sci Med.* (2025) 12:e001363. 10.1136/lupus-2024-001363 39832908 PMC11751792

[4] Ugarte-Gil MF Hanly J Urowitz M Gordon C Bae SC Romero-Diaz J et al. Remission and Low Disease Activity (LDA) prevent damage accrual in patients with systemic lupus erythematosus: results from the Systemic Lupus International Collaborating Clinics (SLICC) inception cohort. *Ann Rheum Dis.* (2022) 81:1541–8. 10.1136/ard-2022-222487 35944946 PMC10353886

[5] Piga M Parodis I Touma Z Legge A Ugarte-Gil MF Hmamouchi I et al. Framework for implementing treat-to-target in systemic lupus erythematosus routine clinical care: consensus statements from an international task force. *Autoimmun Rev.* (2025) 24:103773. 10.1016/j.autrev.2025.103773 39961575

[6] Fanouriakis A Tziolos N Bertsias G Boumpas DT. Update *o*n the diagnosis and management of systemic lupus erythematosus. *Ann Rheum Dis.* (2021) 80:14–25. 10.1136/annrheumdis-2020-218272 33051219

[7] Morand EF Fernandez-Ruiz R Blazer A Niewold TB. Advances in the management of systemic lupus erythematosus. *BMJ.* (2023) 383:e073980. 10.1136/bmj-2022-073980 37884289

[8] Lü LJ Teng JL Bao CD Han XH Sun LY Xu JH et al. Safety and efficacy of T-614 in the treatment of patients with active rheumatoid arthritis: a double blind, randomized, placebo-controlled and multicenter trial. *Chin Med J.* (2008) 121:615–9. 18466681

[9] Lu LJ Bao CD Dai M Teng JL Fan W Du F et al. Multicenter, randomized, double-blind, controlled trial of treatment of active rheumatoid arthritis with T-614 compared with methotrexate. *Arthritis Rheum.* (2009) 61:979–87. 10.1002/art.24643 19565542

[10] Hara M Abe T Sugawara S Mizushima Y Hoshi K Irimajiri S et al. Efficacy and safety of iguratimod compared with placebo and salazosulfapyridine in active rheumatoid arthritis: a controlled, multicenter, double-blind, parallel-group study. *Mod Rheumatol.* (2007) 17:1–9. 10.1007/s10165-006-0542-y 17278015

[11] Hara M Ishiguro N Katayama K Kondo M Sumida T Mimori T et al. Safety and efficacy of combination therapy of iguratimod with methotrexate for patients with active rheumatoid arthritis with an inadequate response to methotrexate: an open-label extension of a randomized, double-blind, placebo-controlled trial. *Mod Rheumatol.* (2014) 24:410–8. 10.3109/14397595.2013.843756 24252050

[12] Kang Y Yan Q Fu Q Wang R Dai M Du F et al. Iguratimod as an alternative induction therapy for refractory lupus nephritis: a preliminary investigational study. *Arthritis Res Ther.* (2020) 22:65. 10.1186/s13075-020-02154-7 32228698 PMC7106733

[13] Yan Q Zhang M Du F Kang Y Ye P Li Q et al. Efficacy and safety of Iguratimod as an add-on therapy for refractory lupus nephritis: a preliminary investigational study. *Front Immunol.* (2023) 14:1062919. 10.3389/fimmu.2023.1062919 36969217 PMC10030952

[14] Yan Q Du F Huang X Fu Q Chen S Dai D et al. Prevention of immune nephritis by the small molecular weight immunomodulator iguratimod in MRL/lpr mice. *PLoS One.* (2014) 9:e108273. 10.1371/journal.pone.0108273 25271634 PMC4182720

[15] Xia Y Fang X Dai X Li M Jin L Tao J et al. Iguratimod ameliorates nephritis by modulating the Th17/Treg paradigm in pristane-induced lupus. *Int Immunopharmacol.* (2021) 96:107563. 10.1016/j.intimp.2021.107563 33812258

[16] Petri M Orbai AM Alarcón GS Gordon C Merrill JT Fortin PR et al. Derivation and validation of the systemic lupus international collaborating clinics classification criteria for systemic lupus erythematosus. *Arthritis Rheum.* (2012) 64:2677–86. 10.1002/art.34473 22553077 PMC3409311

[17] Yan Q Du F Kang Y Ye P Wang X Xu J et al. Comparison of iguratimod and conventional cyclophosphamide with sequential azathioprine as treatment of active lupus nephritis: study protocol for a multi-center, randomized, controlled clinical trial (iGeLU study). *Trials.* (2021) 22:530. 10.1186/s13063-021-05475-3 34380536 PMC8356213

[18] Djabarouti S Breilh D Duffau P Lazaro E Greib C Caubet O et al. Steady-state mycophenolate mofetil pharmacokinetic parameters enable prediction of systemic lupus erythematosus clinical flares: an observational cohort study. *Arthritis Res Ther.* (2010) 12:R217. 10.1186/ar3202 21176194 PMC3046530

[19] Miyazaki Y Nakayamada S Sonomoto K Kawabe A Inoue Y Okubo N et al. Efficacy and safety of belimumab during maintenance therapy in patients with systemic lupus erythematosus. *Rheumatology.* (2022) 61:3614–26. 10.1093/rheumatology/keab953 34962998 PMC9434316

[20] van Vollenhoven RF Bertsias G Doria A Isenberg D Morand E Petri MA et al. 2021 DORIS definition of remission in SLE: final recommendations from an international task force. *Lupus Sci Med.* (2021) 8:e000538. 10.1136/lupus-2021-000538 34819388 PMC8614136

[21] Franklyn K Lau CS Navarra SV Louthrenoo W Lateef A Hamijoyo L et al. Definition and initial validation of a Lupus Low Disease Activity State (LLDAS). *Ann Rheum Dis.* (2016) 75:1615–21. 10.1136/annrheumdis-2015-207726 26458737

[22] Bai Y Zhao J Wang Q Xu D Zeng X Tian X et al. Prediction of flares in systemic lupus erythematosus during post-remission follow-up. *J Inflamm Res.* (2025) 18:3377–84. 10.2147/JIR.S504995 40070926 PMC11895687

[23] van Vollenhoven RF Mosca M Bertsias G Isenberg D Kuhn A Lerstrøm K et al. Treat-to-target in systemic lupus erythematosus: recommendations from an international task force. *Ann Rheum Dis.* (2014) 73:958–67. 10.1136/annrheumdis-2013-205139 24739325

[24] Yang Z Cheng C Wang Z Wang Y Zhao J Wang Q et al. Prevalence, predictors, and prognostic benefits of remission achievement in patients with systemic lupus erythematosus: a systematic review. *Arthritis Care Res.* (2022) 74:208–18. 10.1002/acr.24464 32986933

[25] Ugarte-Gil MF Gamboa-Cardenas RV Reátegui-Sokolova C Pimentel-Quiroz VR Medina M Elera-Fitzcarrald C et al. LLDAS (lupus low disease activity state) and/or remission are associated with less damage accrual in patients with systemic lupus erythematosus from a primarily Mestizo population: data from the Almenara Lupus Cohort. *Lupus Sci Med.* (2022) 9:e000616. 10.1136/lupus-2021-000616 35193948 PMC8867305

[26] Golder V Kandane-Rathnayake R Li N Louthrenoo W Chen YH Cho J et al. Association of sustained lupus low disease activity state with improved outcomes in systemic lupus erythematosus: a multinational prospective cohort study. *Lancet Rheumatol.* (2024) 6:e528–36. 10.1016/S2665-9913(24)00121-8 38876129

[27] Sammaritano LR Askanase A Bermas BL Dall’Era M Duarte-García A Hiraki LT et al. American College of Rheumatology (ACR) guideline for the treatment of systemic lupus erythematosus. *Arthritis Care Res.* (2025). 10.1002/acr.25690 [Epub ahead of print]. 41182321

[28] Fei Y Zhao L Wu L Zuo X Li R Cheng J et al. Evaluation and prediction of relapse risk in stable systemic lupus erythematosus patients after glucocorticoid withdrawal (PRESS): an open-label, multicentre, non-inferiority, randomised controlled study in China. *Ann Rheum Dis.* (2025) 84:274–83. 10.1136/ard-2024-225826 39919900

[29] Gao D Ji L Zhang X Hao Y Xie W Fan Y et al. Transitioning from lupus low disease activity state to remission in systemic lupus erythematosus: real-world evidence. *Front Immunol.* (2025) 16:1546306. 10.3389/fimmu.2025.1546306 40181969 PMC11965674

[30] Barber MRW Drenkard C Falasinnu T Hoi A Mak A Kow NY et al. Global epidemiology of systemic lupus erythematosus. *Nat Rev Rheumatol.* (2021) 17:515–32. 10.1038/s41584-021-00668-1 34345022 PMC8982275

