# Supplementary material for: The efficacy and safety of iguratimod treatment in stable systemic lupus erythematosus: a preliminary prospective cohort study
Source: Front Med (Lausanne). 2026 Jun 1;13:1810291. doi: 10.3389/fmed.2026.1810291 (PMC13265272; doi:10.3389/fmed.2026.1810291)
Supplement: Supplementary file 1 [file Data_Sheet_1.pdf]

# Supplementary materials

Table S1. Baseline characters and outcomes of control group. ........................................................2
Table S2. Analysis of the SLEDAI-2K and glucocorticoid dosage ................................................ 6
Table S3. Changes of serologic indicators at week 26. .......................................................................7
Figure S1. The proportion of patients of the two groups experiencing a treatment failure. ..............8
Table S4. STROBE Statement—Checklist of items ....................................................................... 9

**Table S1. Baseline characters and outcomes of control group**

| Patient no. | Age | Sex | Duration* (Y) | Antibodies$^{\Delta}$ | Organ involvement | IS AND IMM# | Pre-enrollment stability period (w)& | Prednisone dose§ | SLEDAI-2K** | Duration of time (w)$ | Outcome at the last visit |
|---|---|---|---|---|---|---|---|---|---|---|---|
| 1 | 53 | F | 1 | Sm, Ro-52, U1-RNP | Musculoskeletal | AZA、HCQ | 12 | 10 | 0 | 104 | Follow-up, prednisone reduced to 5mg |
| 2 | 53 | F | 2 | Sm, SSA, Rib-P | Hematologic, Gastrointestinal, Renal | MMF, HCQ | 20 | 5 | 2 | 26 | Disease fluctuation, proteinuria; added CsA |
| 3 | 37 | F | 2 | Sm, U1-RNP, Rib-P | Renal, Hematologic,Musculoskeletal | MMF, HCQ | 24 | 5 | 0 | 204 | Follow-up |
| 4 | 34 | F | 2 | Sm, U1-RNP, dsDNA, Nuc, Rib-P | Renal, Hematologic,Musculoskeletal | MMF, HCQ | 30 | 5 | 3 | 64 | Disease fluctuation, proteinuria; added CsA and prednisone increased to 10mg |
| 5 | 30 | F | 0.5 | U1-RNP, Histone, Nuc | Mucocutaneous | HCQ | 20 | 10 | 0 | 152 | Follow-up |
| 6 | 38 | F | 7 | Sm, Ro-52 | Mucocutaneous | HCQ, MTX | 54 | 10 | 0 | 100 | Stable but poor compliance |
| 7 | 47 | F | 6 | Sm, U1-RNP, Histone, Nuc, Rib-P | Musculoskeletal, Hematologic, respiratory | MMF, HCQ | 32 | 5 | 3 | 12 | Worsening arthritis with pulmonary infection |
| 8 | 27 | F | 12 | SSA, SSB | Mucocutaneous, Neurological | MMF, HCQ, MTX | 12 | 5 | 0 | 34 | Seizure recurrence |
| 9 | 31 | F | 8 | Sm, SSA, SSB, U1-RNP | Musculoskeletal, Mucocutaneous | MMF, HCQ, | 32 | 0 | 4 | 42 | Rash flare-up |

| Patient no. | Age | Sex | Duration* (Y) | Antibodies^Δ | Organ involvement | IS AND IMM# | Pre-enrollment stability period (w)& | Prednisone dose§ | SLEDAI-2K** | Duration of time (w)$ | Outcome at the last visit |
|---|---|---|---|---|---|---|---|---|---|---|---|
| 10 | 49 | F | 14 | SSA, SSB, RO-52 | Musculoskeletal, Mucocutaneous, Renal | HCQ、MMF | 12 | 5 | 0 | 40 | Active disease with UTI; switched to CsA |
| 11 | 35 | F | 1 | SSA | Hematologic, Musculoskeletal, Mucocutaneous, | HCQ,MMF | 24 | 7.5 | 2 | 108 | Arthritis Flare-up |
| 12 | 56 | F | 22 | Sm, SSA | Mucocutaneous, Renal | CsA, HCQ | 44 | 10 | 4 | 172 | Follow-up，prednisone reduced to 5mg |
| 13 | 27 | F | 0.5 | SSA, RO-52, dsDNA | Musculoskeletal, Mucocutaneous | CsA, HCQ | 12 | 15 | 2 | 148 | Follow-up，prednisone reduced to 5mg |
| 14 | 30 | F | 1 | Sm, SSA, dsDNA | Musculoskeletal, Mucocutaneous | CsA, HCQ | 14 | 7.5 | 4 | 128 | Rash flare-up; switched to AZA, prednisone increased to 10mg |
| 15 | 60 | F | 2 | Sm, U1RNP | Musculoskeletal | HCQ, MTX | 12 | 0 | 0 | 120 | Follow-up |
| 16 | 55 | F | 1 | U1RNP, SSA | Musculoskeletal | MMF, HCQ | 14 | 5 | 4 | 16 | MMF intolerance; switched to MTX |
| 17 | 66 | F | 16 | SSB, SSA, RO-52, Nuc | Musculoskeletal | MTX, HCQ | 16 | 10 | 2 | 124 | Follow-up |
| 18 | 36 | F | 4 | Sm, U1RNP, SSA | Musculoskeletal | CsA, HCQ | 16 | 0 | 1 | 108 | Follow-up |
| 19 | 45 | F | 2 | Sm, U1RNP, SSA, RO-52 | Musculoskeletal, Mucocutaneous | AZA, HCQ, MTX | 100 | 5 | 0 | 104 | Rash flare-up; MMF initiated |

| Patient no. | Age | Sex | Duration[*] (Y) | Antibodies[Δ] | Organ involvement | IS AND IMM[#] | Pre-enrollment stability period (w)[&] | Prednisone dose[§] | SLEDAI-2K[**] | Duration of time (w)[$] | Outcome at the last visit |
|---|---|---|---|---|---|---|---|---|---|---|---|
| 20 | 35 | F | 0.5 | Sm, U1RNP, SSA, RO-52 | Musculoskeletal | MXT, HCQ | 12 | 0 | 4 | 8 | MTX intolerance; switched to AZA |
| 21 | 53 | F | 1 | Sm, U1RNP | Musculoskeletal, Mucocutaneous | MXT, HCQ | 16 | 7.5 | 2 | 26 | Rash flare-up; switched to azathioprine |
| 22 | 31 | F | 9 | Sm, SSB, U1RNP, SSA | Musculoskeletal, Mucocutaneous | MMF, HCQ | 80 | 0 | 2 | 164 | Rash flare-up; added prednisone |
| 23 | 58 | F | 2 | Rib-P | Musculoskeletal, Mucocutaneous | MMF, HCQ | 12 | 0 | 4 | 110 | Follow-up |
| 24 | 36 | F | 0.5 | U1RNP, SSA, RO-52, Nuc | Musculoskeletal, Mucocutaneous | AZA, HCQ | 28 | 10 | 4 | 148 | Follow-up; prednisone taper |
| 25 | 47 | F | 13 | Sm, U1RNP, SSA, | Musculoskeletal, Mucocutaneous | MMF, MTX, HCQ | 44 | 7.5 | 2 | 58 | Follow-up; prednisone taper |
| 26 | 32 | F | 0.5 | Sm, U1RNP | Hematologic, respiratory, Musculoskeletal | MMF, HCQ | 12 | 5 | 4 | 112 | Follow-up; prednisone taper |
| 27 | 42 | F | 13 | Sm, U1RNP, SSA, RO-52, | Mucocutaneous, Musculoskeletal | MMF, HCQ | 68 | 10 | 2 | 58 | Follow-up; prednisone taper |
| 28 | 40 | F | 1 | Sm, SSA, U1RNP, Rib-P | Mucocutaneous, Musculoskeletal | CsA, HCQ | 32 | 0 | 0 | 120 | Follow-up |
| 29 | 48 | F | 11 | U1RNP | Mucocutaneous, Musculoskeletal | MTX, TGT, HCQ | 52 | 5 | 4 | 48 | Disease fluctuatio; proteinuria |

| Patient no. | Age | Sex | Duration[*] (Y) | Antibodies[Δ] | Organ involvement | IS AND IMM[#] | Pre-enrollment stability period (w)[&] | Prednisone dose[§] | SLEDAI-2K[**] | Duration of time (w)[$] | Outcome at the last visit |
|---|---|---|---|---|---|---|---|---|---|---|---|
| 30 | 37 | F | 5 | Sm | Mucocutaneous, Musculoskeletal, Hematologic | LEF, HCQ | 48 | 5 | 0 | 116 | Stable condition; lost t o follow-up |
| 31 | 40 | F | 8 | Sm, U1RNP | Mucocutaneous, Musculoskeletal | MTX, HCQ | 12 | 10 | 3 | 156 | Follow-up, prednisone taper |

* Disease duration was defined as the time from the onset of initial clinical symptoms to study enrollment. Δ Autoantibody status at the time of enrollment. All enrolled patients had a positive antinuclear antibody (ANA) titer of >1:100 (reference value: 1:100). # Use of disease-modifying antirheumatic drugs (DMARDs) and immunomodulators at baseline, with stable doses for at least 12 weeks prior to enrollment. & Duration of stable disease activity, defined as the time from when the SLE Disease Activity Index 2000 (SLEDAI-2K) score was first documented to be ≤ 4 until enrollment. § Dosage of glucocorticoids at baseline, which had been stable for at least 12 weeks prior to enrollment. ** SLEDAI-2K score at baseline. $ The observation period spanned from the date of study enrollment to the date of the last follow-up visit. IS: immunosuppressant; Imm: immunomodulator; HCQ: Hydroxychloroquine; AZA: azathioprine; TGP: total glucosides of white paeony; MMF: mycophenolate mofetil; CsA, cyclosporin A; MTX: methotrexate; CTX: cyclophosphamide; TGT: tripterygium glycosides tablet; LEF: leflunomide; UTI: urinary tract infections; IS: Immunosuppressants; IMM: Immunomodulators.

**Table S2. Linear mixed-effects model analysis of the SLEDAI-2K and glucocorticoid dosage at 26 and 52 weeks**

| | Estimate | Std.Error | Sign. | 95% CI | |
|---|---|---|---|---|---|
| | | | | Lower Bound | Upper Bound |
| A<br>SLEDAI-2K<br>Baseline | 0.301 | 0.091 | 0.002 | 0.119 | 0.484 |
| A<br>SLEDAI-2K<br>Group (IGU vs Control) | 0.281 | 0.370 | 0.454 | -0.475 | 1.038 |
| A<br>SLEDAI-2K<br>Intercept (group * time) | -0.232 | 0.624 | 0.711 | -1.481 | 1.016 |
| B<br>Glucocorticoid dosage<br>Baseline | 0.555 | 0.083 | <0.001 | 0.389 | 0.722 |
| B<br>Glucocorticoid dosage<br>Group (IGU vs Control) | -0.134 | 1.181 | 0.910 | -2.546 | 2.278 |
| B<br>Glucocorticoid dosage<br>Intercept (group * time) | -0.354 | 1.530 | 0.818 | -3.414 | 2.706 |

**Table S3. Changes of serologic indicators at week 26**

| | IGU group (n=16) | | | Control group (n=31) | | | $P^b$ | | | $P^d$ |
|---|---|---|---|---|---|---|---|---|---|---|
| | Baseline | Final | Change from baseline | Baseline | Final | Change from baseline | Unadjusted between-group difference in change [a] | | Baseline adjusted between-group difference in change [c] | |
| C3 (g/L) | 0.97±0.19 | 0.97±0.19 | 0.00 (-0.90, 0.03) | 0.93±0.21 | 0.91±0.19 | -0.02 (-0.13, 0.03) | 0.02 (-0.02, 0.04) | 0.522 | 0.02 (-0.04, 0.09) | 0.476 |
| C4 (g/L) | 0.17±0.04 | 0.18±0.04 | 0.00 (-0.02, 0.02) | 0.15±0.08 | 0.16±0.09 | -0.01 (-0.02, 0.02) | 0.01 (-0.03, 0.07) | 0.726 | 0.01 (-0.01, 0.04) | 0.396 |
| IgA (g/L) | 2.34 (2.06, 3.97) | 2.28 (1.74, 3.85) | -0.01 (-0.88, 0.39) | 2.82 (2.06, 3.62) | 2.70 (2.08, 3.72) | 0.02 (-0.17, 0.20) | -0.03 (-0.22, 0.05) | 0.234 | -0.09 (-0.48, 0.30) | 0.608 |
| IgG (g/L) | 13.11±4.19 | 15.75±9,61 | 0.37 (-1.59, 8.04) | 17.72±8.65 | 16.79±6.91 | 0.04 (-1.39, 1.09) | 0.33 (-1.89, 0.56) | 0.284 | 0.20 (-0.65, 1.05) | 0.628 |
| IgM (g/L) | 0.83, (0.56, 0.99) | 0.85 (0.56, 1.27) | 0.05 (-0.42, 0.35) | 0.82 (0.64, 1.07) | 0.83 (0.56, 1.14) | -0.01 (-0.09, 0.17) | 0.06 (-0.10, 0.04) | 0.432 | 0.01 (-0.18, 0.21) | 0.882 |

a: Date are median (95% CI). b: *p* value from Mann-Whitney U test. c:Levels of IgA, IgG, and IgM were analyzed using square-root-transformed data and are summarized as mean (95% CI). d Treatment difference and *p*-value derived from an analysis of covariance (ANCOVA) model, with adjustment for baseline values. C3: Complement Component 3, C4: Complement Component 4, IgA: Immunoglobulin A, IgG: Immunoglobulin G, IgM:Immunoglobulin M.

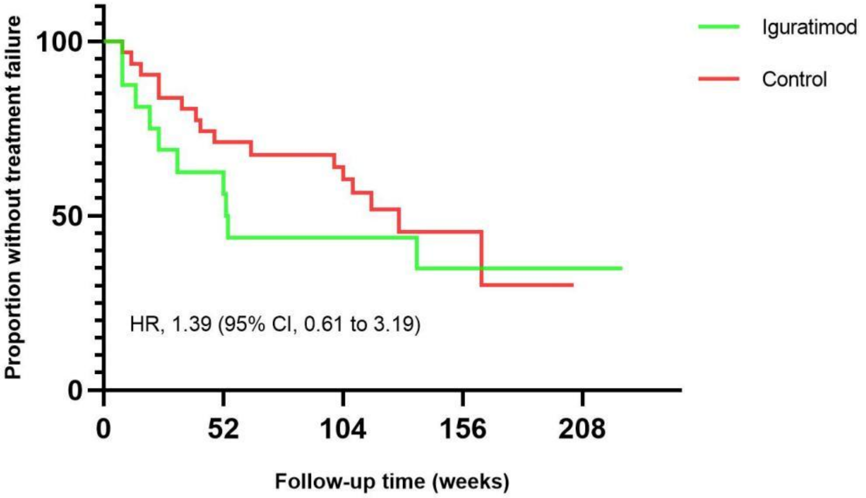


**Figure S1. The Kaplan-Meier survival curve showing the proportion of systemic lupus erythematosus patients of the two groups experiencing a treatment failure over study period.**

**STROBE Statement—Checklist of items that should be included in reports of cohort studies**

| | Item No | Recommendation | Self-check |
|---|---|---|---|
| **Title and abstract** | 1 | (*a*) Indicate the study's design with a commonly used term in the title or the abstract | √ |
| | | (*b*) Provide in the abstract an informative and balanced summary of what was done and what was found | √ |
| **Introduction** | | | |
| Background/rationale | 2 | Explain the scientific background and rationale for the investigation being reported | √ |
| Objectives | 3 | State specific objectives, including any prespecified hypotheses | √ |
| **Methods** | | | |
| Study design | 4 | Present key elements of study design early in the paper | √ |
| Setting | 5 | Describe the setting, locations, and relevant dates, including periods of recruitment, exposure, follow-up, and data collection | √ |
| Participants | 6 | (*a*) Give the eligibility criteria, and the sources and methods of selection of participants. Describe methods of follow-up | √ |
| | | (*b*) For matched studies, give matching criteria and number of exposed and unexposed | √ |
| Variables | 7 | Clearly define all outcomes, exposures, predictors, potential confounders, and effect modifiers. Give diagnostic criteria, if applicable | √ |
| Data sources/ measurement | 8* | For each variable of interest, give sources of data and details of methods of assessment (measurement). Describe comparability of assessment methods if there is more than one group | √ |
| Bias | 9 | Describe any efforts to address potential sources of bias | √ |
| Study size | 10 | Explain how the study size was arrived at | √ |
| Quantitative variables | 11 | Explain how quantitative variables were handled in the analyses. If applicable, describe which groupings were chosen and why | √ |
| Statistical methods | 12 | (*a*) Describe all statistical methods, including those used to control for confounding | √ |

| | | | |
|---|---|---|---|
| | | (*b*) Describe any methods used to examine subgroups and interactions | √ |
| | | (*c*) Explain how missing data were addressed | √ |
| | | (*d*) If applicable, explain how loss to follow-up was addressed | √ |
| | | (*e*) Describe any sensitivity analyses | √ |
| **Results** | | | |
| Participants | 13* | (a) Report numbers of individuals at each stage of study—eg numbers potentially eligible, examined for eligibility, confirmed eligible, included in the study, completing follow-up, and analysed | √ |
| | | (b) Give reasons for non-participation at each stage | √ |
| | | (c) Consider use of a flow diagram | √ |
| Descriptive data | 14* | (a) Give characteristics of study participants (eg demographic, clinical, social) and information on exposures and potential confounders | √ |
| | | (b) Indicate number of participants with missing data for each variable of interest | √ |
| | | (c) Summarise follow-up time (eg, average and total amount) | √ |
| Outcome data | 15* | Report numbers of outcome events or summary measures over time | √ |
| Main results | 16 | (*a*) Give unadjusted estimates and, if applicable, confounder-adjusted estimates and their precision (eg, 95% confidence interval). Make clear which confounders were adjusted for and why they were included | √ |
| | | (*b*) Report category boundaries when continuous variables were categorized | √ |
| | | (*c*) If relevant, consider translating estimates of relative risk into absolute risk for a meaningful time period | Not applicable |
| Other analyses | 17 | Report other analyses done—eg analyses of subgroups and interactions, and sensitivity analyses | √ |
| **Discussion** | | | |
| Key results | 18 | Summarise key results with reference to study objectives | √ |
| Limitations | 19 | Discuss limitations of the study, taking into account sources of potential bias or imprecision. Discuss both direction and magnitude of any potential bias | √ |

| | | | |
|---|---|---|---|
| Interpretation | 20 | Give a cautious overall interpretation of results considering objectives, limitations, multiplicity of analyses, results from similar studies, and other relevant evidence | √ |
| Generalisability | 21 | Discuss the generalisability (external validity) of the study results | √ |
| **Other information** | | | |
| Funding | 22 | Give the source of funding and the role of the funders for the present study and, if applicable, for the original study on which the present article is based | √ |

*Give information separately for exposed and unexposed groups.

**Note:** An Explanation and Elaboration article discusses each checklist item and gives methodological background and published examples of transparent reporting. The STROBE checklist is best used in conjunction with this article (freely available on the Web sites of PLoS Medicine at http://www.plosmedicine.org/, Annals of Internal Medicine at http://www.annals.org/, and Epidemiology at http://www.epidem.com/). Information on the STROBE Initiative is available at http://www.strobe-statement.org.
